# Lower serum interleukin‐22 and interleukin‐35 levels are associated with disease status in neuromyelitis optica spectrum disorders

**DOI:** 10.1111/cns.13198

**Published:** 2019-07-24

**Authors:** Hong Yang, Lu Han, Yun‐Jia Zhou, Jie Ding, Yu Cai, Rong‐Hua Hong, Yong Hao, De‐Sheng Zhu, Xia‐Feng Shen, Yang‐Tai Guan

**Affiliations:** ^1^ Department of Neurology, The First Rehabilitation Hospital of Shanghai, School of Medicine Tong Ji University Shanghai China; ^2^ Department of Neurology, Renji Hospital, School of Medicine Shanghai Jiao Tong University Shanghai China

**Keywords:** immunosuppression, interleukin‐22, interleukin‐35, neuromyelitis optica spectrum disorders

## Abstract

**Aims:**

The exact pathogenesis of neuromyelitis optica spectrum disorder (NMOSD) remains unclear. A variety of cytokines are involved, but few studies have been performed to explore the novel roles of interleukin‐22 (IL‐22) and interleukin‐35 (IL‐35) in NMOSD. Therefore, this study was designed to investigate serum levels of IL‐22 and IL‐35, and their correlations with clinical and laboratory characteristics in NMOSD.

**Methods:**

We performed a cross‐section study, 18 patients with acute NMOSD, 23 patients with remission NMOSD, and 36 healthy controls were consecutively enrolled. Serum levels of IL‐22 and IL‐35 were measured by enzyme‐linked immunosorbent assay (ELISA). The correlations between serum IL‐22 and IL‐35 levels and clinical and laboratory characteristics were evaluated by Spearman's rank or Pearson's correlation coefficient.

**Results:**

The serum levels of IL‐22 and IL‐35 were significantly lower in patients with acute NMOSD and remission NMOSD than in healthy controls (IL‐22: 76.96 ± 13.62 pg/mL, 87.30 ± 12.79 pg/mL, and 94.02 ± 8.52 pg/mL, respectively, *P* < .0001; IL‐35: 45.52 ± 7.04 pg/mL, 57.07 ± 7.68 pg/mL, and 60.05 ± 20.181 pg/mL, respectively, *P* < .0001). Serum levels of IL‐35 were negatively correlated with EDSS scores and cerebrospinal fluid protein levels (*r* = −.5438, *P* = .0002 and *r* = −.3523, *P* = .0258, respectively) in all patients.

**Conclusions:**

Lower serum levels of IL‐22 and IL‐35 are associated with disease status in NMOSD. Additionally, lower serum levels of IL‐35 are associated with disease severity in NMOSD.

## 1 INTRODUCTION

Neuromyelitis optica spectrum disorder (NMOSD) has become one of the most rapidly diagnosed and progressing diseases in the field of neurology in the past 10 years. The median age of onset is 39 years old, and it is much more common among women than men (reported ratios range from 3:1 to 9:1).^1^, ^2^ It was formerly known as optic neuromyelitis or Devic disease. A specific antibody, NMO‐IgG, binds to aquaporin‐4 (AQP4) at the synaptic ends of astrocytes located on the blood‐brain barrier (BBB), resulting in the loss of myelin.^3^ In 2015, the Wingerchuk^4^ group released the first NMOSD diagnostic standard. With the determination of the latest diagnostic criteria for NMOSD, more and more NMOSDs are being recognized. However, the exact pathogenesis of NMOSD remains unclear. Many recent studies have shown that a variety of cytokines and inflammatory chemokines are involved in autoimmune responses as well as central or peripheral nervous system damage; these include IL‐17A, IL‐6, C‐X‐C motif chemokine ligand (CXCL8), and CXCL10.^5^ However, few studies have explored the roles of interleukin‐22 (IL‐22) and interleukin‐35 (IL‐35) in NMOSD.

Interleukin‐22 belongs to the IL‐10 cytokine family but has unique traits that make it different from IL‐10. A broad variety of lymphocytes secrete IL‐22, including Th17, Th22, and NK cells, neutrophils, and innate lymphoid cells.^6^ The biological effects of IL‐22 are mediated by the IL‐22‐IL‐22R pathway.^7^ Its involvement has been reported in many autoimmune diseases, including protective, pathogenic, and dual effects. In protective effects, a decrease in IL‐22 may be considered a risk factor for type 2 diabetes and could worsen hepatitis.^8^, ^9^ However, in other diseases, IL‐22 could be pathogenic. For example, in rheumatoid arthritis, patients who responded to treatment showed reductions in plasma IL‐22 levels, indicating that plasma IL‐22 might play a detrimental role in this disease.^10^ In psoriasis, IL‐22 downregulates Cx43 expression, which could lead to keratinocyte hyperproliferation.^11^ Interestingly, in some diseases, IL‐22 plays a dual role. In systemic lupus erythaematosus (SLE), decreased plasma IL‐22 levels are correlated with SLE disease activity and could result in the rs2227513 polymorphism, which might contribute to SLE susceptibility.^12^, ^13^ In contrast, the Yang^14^ group showed that IL‐22 levels in both the serum and the kidneys were significantly higher in lupus nephritis (LN) patients than in healthy controls. Considering the complexity of IL‐22, we determined that identifying its role in NMOSD might be slightly challenging.

Interleukin‐35 is the newest identified anti‐inflammatory cytokine, which belongs to the interleukin‐12 cytokine family, a unique group of heterodimeric cytokines that include IL‐12, IL‐23, IL‐27, and IL‐35.^15^ Intracellular signaling is mediated by the activation of the JAK‐STAT pathway.^16^ In patients with conditions such as ulcerative colitis, Crohn's disease, primary Sjogren's syndrome, and chronic obstructive pulmonary disease (COPD), the serum levels of IL‐35 are low.^17^, ^18^, ^19^, ^20^ Mesenchymal stem cells with IL‐35 overexpression had stronger immunosuppressive effects than were exerted by non‐transfected mesenchymal stem cells.^21^ Wang^22^ found that treating experimental autoimmune uveitis (EAU) with IL‐35 suppressed uveitis by inhibiting Th17 and Th1 differentiation and inducing the production of regulatory Breg/IL‐35^+^ Breg cells, as well as Tregs. Data from the above studies demonstrate that IL‐35 has an immunosuppressive function in inflammation and that it might be a new target therapy for autoimmune diseases. As NMOSD has very high disability and relapse rates, some patients do not respond to current therapies. It is therefore urgent for us to find new therapies. However, few studies have explored the exact role of IL‐35 in NMOSD.

Therefore, in this study, to improve our understanding of IL‐22 and IL‐35 interrelationships and immunopathologic roles in NMOSD, we investigated the serum levels of IL‐22 and IL‐35 and their correlations with clinical and laboratory characteristics.

## 2 METHODS

### 2.1 Study population

This was a cross‐section study, which was carried out at Shanghai Renji Hospital affiliated with Shanghai Jiao Tong University. In all, 41 patients (18 with acute NMOSD and 23 with remission NMOSD) were consecutively recruited from the clinical centre from January 2015 to June 2016. The patients who were included in this study needed to meet the international consensus diagnostic criteria for NMOSDs established by the Wingerchuk^4^ group in 2015. For acute NMOSD, they were required to be drug‐naïve before sampling. Considering that other autoimmune diseases might influence the results, we had already excluded them before they were recruited in our research.

In total, 36 healthy individuals from the Health Care Centre of Renji Hospital (10 men and 26 women) were also enrolled in the study as a control group. There was no acute or chronic sickness in the control subjects.

The Ethics Committee of Renji Hospital approved all of the research procedures, and we obtained informed consent from all of the participants.

### 2.2 Clinical assessment

Using the hospital's electronic medical record review system, we collected clinical data related to NMOSD patients, including gender, age, the total duration of the disease, the current duration of the disease, the first episode, the first episode diagnosis, the annualized relapse rate (ARR), the serum autoimmune antibodies, the expanded disability status scale (EDSS) score, the presentation of optic neuritis (ON), area postrema symptoms, longitudinally extensive transverse myelitis (LETM), the presence of characteristic spinal cord lesions and brain lesions on MR images, treatment used at the time of sampling, lymphocyte subsets (CD3, CD4, CD8, CD19, CD4/CD8, and NK cells), titers of anti‐AQP4 antibodies, cerebrospinal fluid (CSF) protein levels, immunoglobulin G (IgG) levels, the IgG index, the 24‐hour intrathecal IgG synthesis rate and other biochemical indicators as well as head MRI, cervical MRI, and lumbar MRI results. Regarding these data, the period from the time of the current attack to the time of sampling was regarded as the current disease duration. If the current disease duration was <30 days, the patient was defined as being in the acute phase of the attack. If the current disease duration was more than 30 days, the patient was defining as being in the remission phase of the attack. The period from the time of the first attack to the time of sampling was regarded as the total disease duration. EDSS scores were assessed at the time of sampling. In addition, considering the effects of drugs, patients in the acute phase were required to have not used immunomodulatory drugs before sampling in this study.

### 2.3 Blood sampling and serum cytokine measurements

Blood samples (3‐5 mL) were collected from peripheral veins into a serum‐separating tube (BD Vacutainer®) at the second day of admission. The blood samples were stored at room temperature for approximately 10‐20 minutes and then centrifuged in a high‐speed table top centrifuge at a speed of approximately 400‐900× *g* for approximately 20 minutes. All serum samples were then properly labeled and stored in a −80°C freezer. Serum levels of IL‐22 and IL‐35 were measured using an enzyme‐linked immunosorbent assay (ELISA) kit according to the manufacturer's instructions (R&D systems).

### 2.4 Statistical analysis

Statistical analyses were performed using SPSS (version 23.0) and the GraphPad Prism (version 7.0) statistical program. The Kolmogorov‐Smirnov *Z* test was used to detect the normality of the data. The count data are expressed as a percentage (%), and the chi‐square test or Fisher's exact test was used for comparisons between two groups. Measurement data with a normal distribution are expressed as the mean ± standard deviation (SD). Student's *t* test was used for comparisons between two groups. One‐way ANOVA was used for comparisons among the three groups (total NMOSD, acute NMOSD, remission NMOSD), and LSD multiple comparisons test was used for comparisons within a group. Measurements of non‐normal distribution were expressed as the median and interquartile range (IQR). The Mann‐Whitney *U* test was used for comparisons between groups. Correlations between continuous variables were analyzed by Spearman's rank correlation coefficient or the Pearson correlation coefficient. A two‐tailed probability value <.05 was considered statistically significant.

## 3 RESULTS

### 3.1 Demographic and clinical features of subjects with NMOSD

A total of 41 patients were enrolled. There were 18 patients in the acute phase and drug‐naïve phase and 23 patients in the remission phase. The mean age was 50.00 ± 14.075 years old in the acute group and 40.65 ± 11.089 years old in the remission group (*P* < .05). The female/male ratio was 11/7 in the acute group and 18/5 in the remission group (*P* = .231). Serum anti‐AQP4 antibodies were positive in 9 (50%) patients in the acute group and 10 (43.48%) patients in the remission group (*P* = .075). There was one patient accompanying with positive anti‐SSA in the acute group, one patient accompanying with positive anti‐SSA and one patient with positive rheumatoid factor (RF) in the remission group (*P* = .702). The current disease duration was 9 (IQR = 11) days in the acute group and 60 (IQR = 50) days in the remission group (*P* < .0001). The total disease duration was 12 (IQR = 26) months in the acute group and 6 (IQR = 37) months in the remission group (*P* = .422). The CSF protein level was 412.00 (IQR = 170) mg/L in the acute group and 336.27 ± 130.404 mg/L in the remission group (*P* = .004). With regard to EDSS scores, in the acute group, the median was 6.00 (IQR = 4), and in the remission group, the median was 2 (IQR = 3; *P* = .007). There were 9 (50%) patients with optic neuritis (ON) in the acute group and 5 (21.73%) such patients in the remission group (*P* = .179). In all, 16 (88.89%) patients in the acute group and 16 (69.56%) patients in the remission group had brain invasion (*P* = .138). Fourteen (77.78%) patients in the acute group and 19 (82.61%) patients in the remission group had cervical spinal cord invasion (*P* = .698). Eight (44.44%) patients in the acute group and 13 (56.52%) patients in the remission group had thoracic spinal cord invasion (*P* = .443). One (5.5%) patient in the acute group and 0 (0.00%) patient in the remission group had lumbar spinal cord invasion (*P* = .901; Table 1).

**Table 1:** Characteristics of subjects with neuromyelitis optica spectrum disorder (NMOSD) and healthy controls

	Acute NMOSD (n = 18)	Remission NMOSD (n = 23)	Total NMOSD (n = 41)	Healthy controls (n = 36)	*P* ^1^‐value	*P* ^2^‐value
Mean age (y)	50.00 ± 14.075	40.65 ± 11.089	44.76 ± 13.187	44.58 ± 13.137	.022*	.954
Sex (female/male, %)	11/7 (61.11, 38.89)	18/5 (78.26, 21.74)	29/12 (70.73, 29.27)	26/10 (72.22, 27.78)	.231	.887
Anti‐AQP4 antibody (positive/negative, n, %)	9/9 (50, 50)	10/13 (43.48, 56.52)	19/22 (46.34, 53.66)		.775	
CSF protein (median, IQR, or mean ± SD; mg/L)	412.00 (170)	336.27 ± 130.404	366.50 (159)		.004**	
Current disease duration (median, IQR; d)	9 (11)	60 (50)	38 (67.50)		<.0001****	
Total disease duration (median, IQR; mo)	12 (26)	6 (37)	12 (33.5)		.422	
Serum autoimmune antibodies (positive, %)	1 (5.5)	2 (8.7)	3 (7.3)		.702	
Anti‐DNA (positive, %)	0 (0)	0 (0)				
Anti‐Sm (positive, %)	0 (0)	0 (0)				
Anti‐SSA (positive, %)	1 (5.5)	1 (4.3)				
Anti‐SSB (positive, %)	0 (0)	0 (0)				
Rheumatoid factor (positive, %)	0 (0)	1 (4.3)				
Optic nerve invasion (n, %)	9 (50)	5 (21.73)	14 (34.15)		.179	
Brain invasion^a^ (n, %)	16 (88.89)	16 (69.56)	32 (78.05)		.138	
Cervical spinal cord invasion (n, %)	14 (77.78)	19 (82.61)	33 (80.49)		.698	
Thoracic spinal cord invasion (n, %)	8 (44.44)	13 (56.52)	21 (51.22)		.443	
Lumbar spinal cord invasion (n, %)	1 (5.5)	0 (0)	1 (2.44)		.901	
EDSS score (median, IQR)	6 (4)	2 (3)	3 (5)		.007**	

Note

*P* ^1^: Comparison between acute NMOSD and remission NMOSD.

*P* ^2^: Comparison between total NMOSD and healthy controls.

Abbreviations: Anti‐AQP4 antibody, anti‐aquaporin‐4 antibody; CSF protein, cerebrospinal fluid protein; EDSS score, expanded disability status scale score; IQR, interquartile range; SD, standard deviation.

a Brain invasion: increased signal on T2‐weighted MRI sequence in the patterns defined by Wingerchuk et al.^4^

^*^ *P* < .05, ^**^ *P* < .01, ^***^ *P* < .001, ^****^ *P* < .0001.

We enrolled 36 healthy subjects as controls (mean age, 44.58 ± 13.137 years old, female/male ratio: 26/10), and there were no significant differences between the controls and the total number of patients (mean age, 44.76 ± 13.187 years old; female/male ratio: 29/12) in age and the sex ratio (*P* = .954 and *P* = .887, respectively; Table 1).

### 3.2 Serum levels of IL‐22 and IL‐35 in NMOSD patients and healthy controls

**Figure 1:**
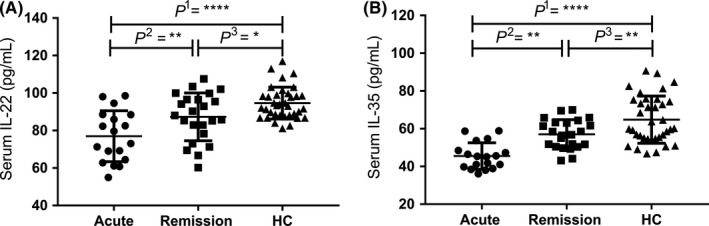
A, Serum interleukin‐22 (IL‐22) levels in patients with neuromyelitis optica spectrum disorder (NMOSD) and healthy controls. Using one‐way ANOVA, we found there were significant differences among the three groups (*P* < .0001). Using LSD multiple comparisons test for acute NNOSD and HC, acute NMOSD and remission NMOSD, and remission NMOSD and HC, we found there were significant differences (*P* ^1^ < .0001, *P* ^2^ < .01, and *P* ^3^ < .05, respectively). B, Serum interleukin‐35 (IL‐35) levels in patients with NMOSD and healthy controls. Using one‐way ANOVA, we show there were significant differences among the three groups (*P* < .0001). Using LSD multiple comparisons test for acute NMOSD and HC, acute NMOSD and remission NMOSD, and remission NMOSD and HC, we show there were significant differences (*P* ^1^ < .0001, *P* ^2^ < .01, and *P* ^3^ < .01, respectively; *P < .05; **P < .01; ***P < .001; ****P < .0001)

The serum levels of IL‐22 were significantly lower in patients with acute NMOSD and remission NMOSD than in the healthy controls (HC; 76.96 ± 13.62 pg/mL, 87.30 ± 12.79 pg/mL, and 94.02 ± 8.52 pg/mL, respectively; *P* < .0001). Additionally, there were significant differences between the acute NNOSD and HC groups, between the acute NMOSD and remission NMOSD groups, and between the remission NMOSD and HC groups (*P* ^1^ < .0001, *P* ^2^ < .01, and *P* ^3^ < .05, respectively; Table 2, Figure 1).

**Table 2:** Measurements of interleukin‐22 (IL‐22) and interleukin‐22 (IL‐35) levels

	Acute NMOSD (n = 18)	Remission NMOSD (n = 23)	Healthy controls (n = 36)	*P*‐value	*P* ^1^‐value	*P* ^2^‐value	*P* ^3^‐value
IL‐35 (pg/mL)	45.52 ± 7.04	57.07 ± 7.68	60.05 ± 20.181	<.0001****	<.0001****	<.01**	<.01**
IL‐22 (pg/mL)	76.96 ± 13.62	87.30 ± 12.79	94.02 ± 8.52	<.0001****	<.0001****	<.01**	<.05*

Note

*P*‐value: One‐way ANOVA among groups.

*P* ^1^‐value: LSD multiple comparisons test between acute NMOSD and HC.

*P* ^2^‐value: LSD multiple comparisons test between acute NMOSD and remission NMOSD.

*P* ^3^‐value: LSD multiple comparisons test between remission NMOSD and HC.

Abbreviation: NMSOD, neuromyelitis optica spectrum disorder.

^*^ *P* < .05, ^**^ *P* < .01, ^***^ *P* < .001, ^****^ *P* < .0001.

The serum levels of IL‐35 were significantly lower in patients with acute NMOSD and chronic NMOSD than in the HC (45.52 ± 7.04 pg/mL, 57.07 ± 7.68 pg/mL, and 60.05 ± 20.181 pg/mL, respectively; *P* < .0001). Additionally, there were significant differences between the acute NNOSD and HC groups, the acute NMOSD and remission NMOSD groups, and the remission NMOSD and HC groups (*P* ^1^ < .0001, *P* ^2^ < .01, and *P* ^3^ < .01, respectively; Table 2, Figure 1).

### 3.3 Correlation between serum IL‐22 and IL‐35 levels

**Figure 2:**
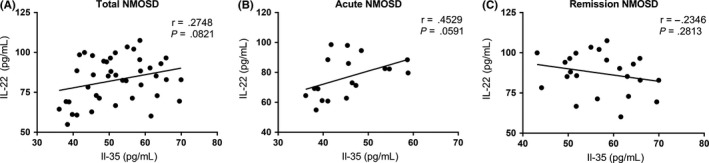
A, B, C, Using the Pearson correlation coefficient, we shown there were no correlations between serum interleukin‐35 (IL‐35) levels and serum interleukin‐22 (IL‐22) levels in the total neuromyelitis optica spectrum disorder (NMOSD), acute NMOSD and remission NMOSD groups (*r* = .2748, *P* = .0821; *r* = .4529, *P* = .0591; and *r* = −.2346, *P* = .2813, respectively)

There were no correlations between serum IL‐22 levels and serum IL‐35 levels among all NMOSD, acute NMOSD, and remission NMOSD individuals (*r* = .2748, *P* = .0821; *r* = .4529, *P* = .0591; and *r* = −.2346, *P* = .2813, respectively; Figure 2).

### 3.4 Correlation between serum levels of IL‐22 and IL‐35 and clinical data in NMOSD

**Figure 3:**
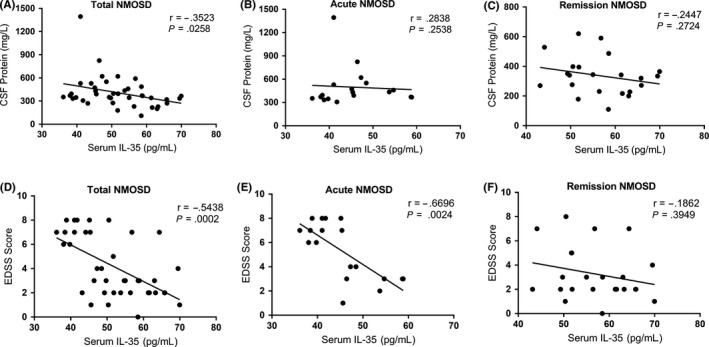
A, Using Spearman's rank correlation coefficient, we show that there was a negative correlation between serum interleukin‐35 (IL‐35) levels and cerebrospinal fluid (CSF) protein levels in the total neuromyelitis optica spectrum disorder (NMOSD) group (*r* = −.3523, *P* = .0258). B, Using the Pearson correlation coefficient, we show there was no correlation between serum IL‐35 levels and CSF protein levels in the total NMOSD group (*r* = .2838, *P* = .2538). C, Using Spearman's rank correlation coefficient, we show there was no correlation between serum IL‐35 levels and CSF protein levels in the remission NMOSD group (*r* = −.2447; *P* = .2724). D, E, F, Using Spearman's rank correlation coefficient, we show there were negative correlations between serum IL‐35 levels and EDSS scores in the total NMOSD and acute NMOSD groups (*r* = −.5438, *P* = .0002; and *r* = −.6696, *P* = .0024; respectively), but there was no correlation in the remission NMOSD group

With regard to serum levels of IL‐35 and EDSS scores, there were significant differences among all NMOSD patients (*r* = −.5438, *P* = .0002) and acute NMOSD patients (*r* = −.6696, *P* = .0024). However, there were no significant differences in the remission NMOSD group (*r* = −.1862, *P* = .3949). For serum IL‐35 levels and CSF protein levels, there were significant differences in the total NMOSD patients (*r* = −.3523, *P* = .0258). However, there were no significant differences in the acute NMOSD and remission NMOSD groups (*r* = −.2838, *P* = .2538; and *r* = −.2447, *P* = .2724, respectively; Figure 3).

**Figure 4:**
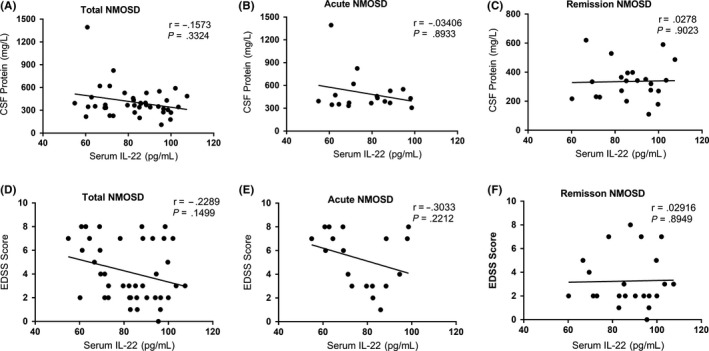
A, B, C, Using Spearman's rank correlation coefficient or Pearson's correlation coefficient, we show there were no correlations between serum interleukin‐22 (IL‐22) levels and cerebrospinal fluid (CSF) protein levels in the total neuromyelitis optica spectrum disorder (NMOSD), acute NMOSD, and remission NMOSD groups (*r* = −.1573, *P* = .3324; *r* = −.03406, *P* = .8933; and *r* = .0278, *P* = .9023, respectively). D, E, F, Using Spearman's rank correlation coefficient, we show there were no correlations between serum IL‐22 levels and EDSS scores in the total NMOSD, acute NMOSD, and remission NMOSD groups (*r* = −.2289, *P* = .1499; *r* = −.3033, *P* = .2212; and *r* = .02916, *P* = .8949, respectively)

With regard to EDSS scores and CSF protein levels, there were no significant differences among the total patients, the acute group, and the remission group in serum IL‐22 levels (Figure 4).

## 4 DISCUSSION

To our knowledge, the serum levels of IL‐22 and IL‐35 have rarely been detected in previous studies of NMOSD. Our study demonstrates that the serum levels of IL‐22 and IL‐35 are dramatically lower in the acute and remission NMOSD groups than in the HC group, indicating that IL‐22 and IL‐35 were related to disease status. The serum levels of IL‐35 were negatively associated with EDSS scores and CSF protein levels in the total NMOSD patients, indicating that IL‐35 was related to disease severity.

Interleukin‐22 is composed of a heterodimer consisting of IL‐22R1 and IL‐10R2. Its original name was IL‐10‐related T cell–derived inducible factor (IL‐TIF), and it was discovered in 2000 as a new member of the IL‐10 family of cytokines.^23^ Various types of cells produce IL‐22, including Th17, Th22, and NK cells, and group 3 innate lymphoid cells (ILC3s).^24^, ^25^, ^26^, ^27^ Recently, increasing evidence has indicated that IL‐22 plays diverse roles in the pathogenesis of autoimmune diseases, including pathogenic and protective processes. To determine whether IL‐22 is abnormally expressed in patients with NMOSD, serum IL‐22 levels were analyzed in patients with NMOSD and in HCs. We found that serum levels of IL‐22 were dramatically lower in the acute and remission NMOSD groups than in the HCs (76.96 ± 13.62 pg/mL, 87.30 ± 12.79 pg/mL, and 94.02 ± 8.52 pg/mL, respectively; *P* < .0001). However, there were no correlations between EDSS scores and CSF protein levels (Table 2, Figure 1, Figure 4). Our findings are opposite to those reported by the Xu group, but this does not mean that our data are mutually exclusive.^28^ Different cell populations produce IL‐22 depending on the phase of disease. Taube et al showed that ILC3s largely contribute to IL‐22 production in allergic airway disease, but Takahashi et al and Ito et al found that CD4^+^ T cells were the main producers of IL‐22 in the same model.^29^, ^30^, ^31^ Similarly, in SLE, Lin et al^12^ found that reduced serum IL‐22 levels might be a distinct feature of new‐onset SLE. Additionally, Wang et al^13^ found that decreasing the expression of IL‐22 might contribute to SLE susceptibility. In contrast, Yang et al^14^ showed that IL‐22 levels were significantly higher in both the serum and kidneys in lupus nephritis (LN) patients than in HCs. These mixed results indicate that IL‐22 exerts bidirectional effects and might play different roles in different phases of diseases. We may need to investigate further reasons. According to the above studies, the fact that our and Xu's group produced different results may be understandable. IL‐22 might play dual roles in mechanisms during different phases of NMOSD. Indeed, some of our patients were in different phases in both of our studies. Xu group just described the total disease duration which was 47.21 ± 46.43 (months).^28^ In our study, the current disease duration was 9(11) (median, IQR; days), the total disease duration was 12 (26) (median, IQR; months) for the acute group, and this may have influenced our results. Additionally, we included more patients and more healthy controls than were included in the Xu's study.

The Yan group found that IL‐22 played a protective role in experimental autoimmune uveitis by converting pathogenic T cells into regulatory T cells.^32^ This finding might partially explain the phenomenon observed in our study. An imbalance between Th17 cells and Treg cells is an important factor underlying NMOSD.^33^, ^34^ Therefore, we hypothesized that a decrease in IL‐22 might result in a decrease in regulatory T cells, which could influence the balance between Th17 cells and Treg cells, thereby resulting in deteriorating NMOSD. IL‐22 may therefore have the potential to be applied as a novel therapy for NMOSD. However, it must be recognized that IL‐22 has a dual nature and further studies are needed to determine the precise cellular sources of IL‐22 in different phases of autoimmune diseases.

IL‐35, a novel anti‐inflammatory cytokine, belongs to the interleukin‐12 cytokine family.^15^ It could inhibit inflammation in many autoimmunity models.^22^, ^35^ To determine whether IL‐35 is abnormally expressed in patients with NMOSD, serum IL‐35 levels were analyzed in patients with NMOSD and HCs. The evidence presented in this study indicates that IL‐35 production is significantly lower in patients with acute and remission NMOSD than in HCs (45.52 ± 7.04 pg/mL, 57.07 ± 7.68 pg/mL, and 60.05 ± 20.181 pg/mL, respectively; *P* < .0001; Table 2, Figure 1), indicating that a reduction in IL‐35 levels may play an important role in regulating NMOSD. Zhang et al^35^ also found similar results in NMOSD, in support of our study. However, in our study, we compared the acute phase of NMOSD to remission NMOSD and HCs. In Zhang's study, there was no remission group. IL‐35 has been confirmed to suppress Teff cell activity and inhibit the differentiation of Th17 cells, and it is critical for regulating the activity of Treg cells.^36^, ^37^ Imbalance between Th17 cells and Treg cells is an important factor underlying NMOSD.^33^, ^34^ Therefore, we speculate that decreased levels of IL‐35 may change the balance between Th17 cells and Treg cells in patients with NMOSD, thereby worsening the symptoms of NMOSD.

In addition, because of findings related to AQP4 antibodies, it is clear that B cell–mediated humoral immunity plays an important role in the pathogenesis of NMOSD.^38^, ^39^, ^40^ In some human autoimmune diseases, there is a lack of equivalent Breg cells with regard to quantity or function.^41^ An interesting finding was that IL‐35 was also correlated with the number of Breg cells.^22^ Wang et al studied Breg cells and IL‐35 cytokines and found that IL‐35 induced Breg cells to produce IL‐10 and IL‐35 (IL‐35^+^ Breg cells). Injection of recombinant IL‐35 or IL‐35^+^ Breg cells into experimental autoimmune uveitis mice inhibited the response of effector T cell 17 (Th17) and effector T cell 1 (Th1) cells and induced Foxp3^+^ Treg cells to improve inflammation.^22^ Therefore, we assumed that the decrease observed in serum IL‐35 levels was correlated with the decrease in Breg cells in our study and that this might indicate that IL‐35 plays a very important role in NMOSD. In addition, we found that serum levels of IL‐35 were negatively correlated with EDSS scores in total NMOSD and remission NMOSD (*r* = −.5438, *P* = .0002; and *r* = −.6696, *P* = .0024, respectively) and negatively correlated with CSF protein levels in total NMOSD (*r* = −.3523, *P* = .0258; Figure 3). As there is currently no known predictor of the severity of NMOSD, IL‐35 might be a meaningful biomarker. Wang et al^22^ also found that recombinant IL‐35 prevented uveitis, while mice lacking IL‐35 developed more severe uveitis. Because some patients do not respond to current therapies, this could also be a promising gene target treatment for NMOSD.

Although a small number of studies have explored the roles of IL‐22 and IL‐35 in the pathogenesis of NMOSD, there are some limitations to our study. For example, we may need to expand the population of enrolled patients. We compared different phases of NMOSD, whereas it would be better to compare data in the same patient between timepoints before and after treatment. In addition, it may be possible to select a highly sensitive method, such as detecting IL‐35 by detecting the levels of the EBI3 and IL‐12p35 mRNAs and detecting IL‐22 by detecting the levels of the IL‐22R1 and IL‐10R2 mRNAs.

## 5 CONCLUSION

In conclusion, we found that serum levels of IL‐22 and IL‐35 were lower in the NMOSD group than in HCs and that serum levels of IL‐35 were negatively associated with EDSS scores and CSF protein levels in total NMOSD patients, indicating that IL‐22 and IL‐35 are associated with disease status; additionally, IL‐35 is associated with disease severity. Therefore, IL‐22 and IL‐35 may have the potential to be applied as novel therapies for NMOSD. However, it must be recognized that IL‐22 has a dual nature. Further studies should be performed to reveal their precise mechanisms in NMOSD.

## CONFLICT OF INTEREST

The authors declare no conflict of interest.
